# Prevalence of rheumatoid arthritis following COVID-19 vaccine: An autoimmune disorder

**DOI:** 10.1016/j.amsu.2022.104628

**Published:** 2022-09-09

**Authors:** Aneesh Rai, Aashish, Priya, Sundiya Karmani, Waseem Abbas, Govinda Khatri

**Affiliations:** Department of Internal Medicine, Dow University of Health Science, Karachi, Pakistan

Rheumatoid arthritis (RA) is symmetric polyarticular arthritis that typically affects the hands and feet's tiny diarthrodial joints. In addition to synovial inflammation, inflammation of the lining of the joint, the forceful front of Pannus tissue invades and destroys local articular tissue structures. The synovium is generally a noncellular tissue, with a delicate intimal lining structure in RA, CD4^+^ T cells Lymphocytes, B cells, and macrophages invade the synovium and cause inflammation. Sometimes they form distinct lymphoid aggregates called germinal centers. Rheumatoid factors and perhaps other autoantibodies produce immune complexes that fix complement and release chemotactic factors such as C5a. Inflammatory cells are then recruited along a chemotactic gradient to the rheumatoid joint, where they are activated and contribute to local damage. In particular, neutrophils aggregate in synovial fluid, where they ingest immune complexes and produce proteolytic enzymes [1]. The diagnosis of rheumatoid arthritis is primarily clinical. There is no single test that can completely diagnose rheumatoid arthritis. A complete blood cell count with differential, rheumatoid factor, and erythrocyte sedimentation rate or C-reactive protein should be included in the initial laboratory testing. To rule out viral or crystal-induced arthritis, joint suction may be necessary in rare cases, particularly in monoarticular presentations. To guide drug choices, baseline renal and hepatic function testing are indicated [2]. Anti-cyclic citrullinated peptide antibody has a high specificity and positive predictive value, however, it is present in less than 60% of rheumatoid arthritis patients [3]. The risk factors for Rheumatoid arthritis include Age (risk increases with age), Sex (risk 2–3 times higher in women), Smoking, Genetics (increased risk in people with certain genes), Obesity, and increased risk of remission in pregnancy [4]. One case reported was of a white male, 55 years old, with non-erosive, seropositive rheumatoid arthritis (positive for rheumatoid factor, anti-cyclic citrullinated peptide antibodies, antinuclear antibodies, and anti-Ro antibodies) who had been in clinical remission for more than 2 years and experienced an acute flare 12 h after the second BNT162b2 vaccination [5].

Vaccines are the most important tool in the fight against infectious diseases. Vaccination with mRNA-based vaccines against severe acute respiratory syndrome coronavirus has resulted in effective protection against coronavirus disease (COVID-19). However, effective immune responses against this virus by vaccination are also associated with a wide range of local and systemic adverse effects. Aside from headaches, fatigue, and muscle pain, autoimmune disorders have been reported as the aftereffects of covid-19 vaccination. Immune thrombotic thrombocytopenia, IgA nephropathy, bullous pemphigoid, Guillain-Barré syndrome, autoimmune liver disorders, and systemic lupus erythematosus are a few examples of new-onset autoimmune events following COVID-19 vaccination [6,7]. The formation of specific autoantibodies, molecular mimicry, and the function of specific vaccine adjuvants all appear to play a significant role in the autoimmune phenomenon. But more research is needed to determine whether the link between the COVID-19 vaccine and autoimmune symptoms is a coincidence or intended [8].

There are two possible disease development scenarios of Rheumatoid Arthritis following Covid-19 vaccination: a flare-up of pre-existing RA and the emergence of de novo RA. Terracina et al. reported a case of a 55-year-old male developing Rheumatoid Arthritis flares 12 h after receiving the second dose of the covid-19 vaccine [5]. Watanabe et al. reported a new onset of rheumatoid arthritis in a 53-year-old male 4 weeks after receiving the covid-19 vaccine [9]. One theory for how autoimmunity can develop following vaccination is molecular mimicry, in which similarities between self-peptides and viral peptides can trigger an immune response [10]. Another theory explaining this phenomenon is spike glycoprotein, which myocytes produce after immunization and could function as an antigenic stimulation. The polymer employed to stabilize the lipid nanoparticle composition, polyethylene glycol, is another possible trigger. It has been proposed that this polymer, which has been linked to type I hypersensitivity reactions, could also cause delayed antibody-mediated reactions. The vaccine might also unmask a hypersensitive reaction to an unexplained trigger, like an asymptomatic infection [11]. Toll-like receptors (TLRs) sensing RNA derived from an mRNA-based vaccine can be powerful triggers to produce pro-inflammatory mediators and type I interferons (IFN–I). Therefore, increased pro-inflammatory cytokine and IFN-1 production may contribute to the emergence of autoimmune reactions following COVID-19 vaccination [12].

Rheumatoid arthritis is diagnosed by the presence of anti-cyclic citrullinated peptide antibodies, rheumatoid factor, and high erythrocyte sedimentation rate or C-reactive protein [13]. Even though the risk of autoimmune disease after the Covid-19 vaccine is higher, individuals need to get a second dose of the vaccine because the benefits of immunization far exceed the risks and, we should not forget the enormous benefits of mass vaccination in preventing COVID-19 morbidity and mortality.

Other vaccines, like those against tetanus, influenza, hepatitis B, and rubella, have also been shown to have the potential to cause rheumatoid arthritis [14]. As a result, while vaccines can prevent the emergence of autoimmune disorders caused by infections, autoimmunity can also occur after vaccination. Nevertheless, we believe that vaccination and SARS-CoV-2 are potential RA triggers, Large-scale epidemiological studies in a flare or new onset RA patients are needed to confirm this link. Data from different studies show that in covid vaccine induced Rheumatoid arthritis, the majority of patients were given glucocorticoids, nonsteroidal anti-inflammatory medications, or analgesics, as well as disease-modifying antirheumatic medications (DMARDs) and methylprednisolone [15]. One study demonstrates that the compound betamethasone therapy was effective against reactive arthritis after COVID-19 vaccination [16].

## Ethics statement

The present study includes printed and published information; therefore, formal ethical clearance was not applicable for this study.

## Funding

None.

## Author contribution

All authors meet the inclusion criteria, and all authors read and approved the final version of the manuscript.

## Registration of research studies

1. Name of the registry: N/A
2. Unique Identifying number or registration ID: N/A
3. Hyperlink to your specific registration (must be publicly accessible and will be checked): N/A

## Guarantor

Govinda Khatri, Department of internal medicine, Dow University of Health Science, Karachi, Pakistan Email: govindakhatri550@gmail.com.

## Consent

N/A.

## Declaration of competing interest

The authors declare that there is no conflict of interest.
